# Cancer of the Uterus and Its Rational Treatment*Address before the Tri-State Medical Association at Chattanooga, Tenn., October 28, 1891.

**Published:** 1891-12

**Authors:** G. Wiley Broome

**Affiliations:** St. Louis, Mo.


### Southern Medical Record.

A MONHLY JOURNAL OF MEDICINE AND SURGERY.

VOL. XXI. ATLANTA, GA., DECEMBER, 1891. NO. 12

### Original Articles.

### CANCER OF THE UTERUS AND ITS RATIONAL TREATMENT.*

BY G. WILEY BROOME, M. D., ST. LOUIS, MO.

Surgical science recognizes no impassable boundaries, no limitation of impossible achievment. It laughs at barriers and exultingly transcends them all. The horizon of to-day becomes the standpoint of observation of to-morrow. The rate of progress sometimes may be deemed slow, and by short gradations; but meanwhile it was fortifying the advances laboriously achieved, and gathering forces for stupendous leaps at the appointed time. Some genius in the van seizes the standard of discovery and plants it far into the regions of the before unknown. These marvelous vaults, therefore, were not the result of momentary impulse or of inconsiderate rashness; they were sublime coronations of previous accomplishments; the deductions from the unit to the aggregate; from the simple to the complex, from the particular to the general. Thus, till within a few years, the great cavities of the body were considered hallowed inclosures, into which no profane hand must intrude.

*Address before the Tri-State Medical Association at Chattanooga, Tenn., October 28, 1891.

But in abdominal surgery, from the appointed hour at which the horologue of time rung out the epoch in McDowell's professional career down to the present moment, many brilliant operations have been performed and many of them established as legitimate surgical procedures.

The advances made in practical surgery of the abdomen in the last few months only are truly astounding, and it may be recorded as a fact that the progress of abdominal surgery during that short time has no parallel in history.

In the special condition, for example, of suppurative peritonitis, modern surgery has demonstrated that incision, irrigation and drainage saves nearly all the cases; whereas, under the most skilled medical treatment a recovery was the exception.

The passive uterus is still the victim of remorseless affections, which hurry its possessors to premature graves. "For these, is there no relief?" humanity imploringly inquires. The genius of surgery promptly replies, "There is; at least there is hope," and then throws herself into the breach. Another battle with incurable disease is then triumphantly won. Hysterectomy is devised and performed; woman still lives, and with life, the inestimable boon of health is conferred.

Now, a significant sound breaks upon the ear, announcing the advent of systematic cerebral surgery. The dome of will, thought, reason, intellection is with skilled hands invaded; abscesses are freely opened, tumors removed; depressed fractures of the skull operated upon irrespective of symptoms; the skull is now opened for intracranial traumatic hemorrhage, also for traumatic epilepsy, and a pathway even to the central ventricles is opened; hope again revives, the patient recovers, a joy to self and friends.

Where, then, is the extreme goal at which progress shall cease? There is none, there can be none, so long as human suffering continues. Immortal laurels have been won in the domain of abdominal and thoracic surgery. The cranium and the pelvis offer equal opportunities for eminent distinction; and these are diligently improved by many throughout the world. And 'tis no presumption or conjecture to assert that magnificent results will be evolved. The sphere and the limitations of each are the momentous questions now on trial. Erudite skill and doubtless ignorant audacity will crowd the arena for fame. Infinite benefit will accrue to the suffering, though some lives may be wantonly sacrificed. But there is a legitimate field, midway between the extremes, into which the wise and prudent surgeon may enter, and find ample scope for the highest achievements to patient, to science and to himself.

Since a prevailing rule forbids any one speaker to consume more than a few moments of your time, allow me to present for your consideration one of the subjects which is just now receiving our best thought and attention, and to which I have already referred. I mean the surgical treatment of malignant disease of the uterus, with especial reference to its removal by kolpo-hysterectomy. Within the short time allotted, I can only incidentally refer to some of the phases of the disease. We believe there is no difference of opinion to-day on the point, that there is no other successful treatment for malignant growths than total ablation. Accepting this view as the only alternative, we must next ascertain what is now understood by the term malignancy.

In contemplating innocent and malignant growths the most important distinction, from a practical point of view, which it has been attempted to draw between different classes of new growths is, that which is expressed by saying that they have, or have not the properties called malignant.

This distinction was originally made without reference to structure, on the basis of the physiology of life-history of tumors. Pathologists agree that malignant growths have the following properties :

1. Recurrent—that is, if removed from the organ in which they are growing, they are liable to recur, and sometimes to be followed by the production of other growths of the same kind in other parts of the body.

2. Destroying the part in which they are growing; and immediately fatal consequences are avoided only by the removal of the entire organ.

The analogy, between the process of malignant tumor-growth and that of specific or infective inflammation, has naturally led to the supposition, that some specific poison may be the cause of the former as it is of the latter. It would be rash to say that such a mode of caution is impossible, but at present no such poison has been found to exist in the case of malignant diseases, and little or no evidence has been given, pointing to the probability of the existence of any such virus.

The chief argument in favor of the theory that malignant growths are produced by some specific virus, like those of infective inflammation, such as pyæmia or tubercle, or those of specific fevers, are somewhat as follows. All other alleged causes of tumor growth in general, or of malignant growth in particular, are inadequate to explain the phenomena which we have called local and general infectiousness. The growth and extension of malignant growths do, on the other hand, resemble the corresponding phenomena in the diseases before mentioned. In the one class, as in the other, the morbid process begins at one spot and spreads; in the first instance, by direct contiguity to neighboring parts, afterward, by the lymphatic and blood channels, to distant parts. It must, therefore, be supposed that in all cases some specific excitant of growths is conveyed from one part to another. Sir James Paget supposes that there is, in the first instance, a constitutional infection, of which the growth, called cancer, is the local manifestation. But the more usual and, perhaps, rational way of presenting the parasitic theory, assumes that the disease is local in the first instance, and afterward becomes more or less general.

It has been shown that anthrax, tuberculosis and leprosy have foreign organisms associated with them, and we may be sure that before long many more will be in the same position. We may find the essential principle of syphilis, and can scarcely fail to find those of the infective fevers and malignancy. The discoveries of Woolbridge and Hankin with regard to the chemical products of the anthrax bacillus open a vista to new fields of view, while they show much that is old in a new light. The anthrax bacillus has been shown to engender a substance of the nature of albumose, which is supposed to be fatal to the baccillus itself, while upon the affected animal it has a duplex action; in large doses it produces the symptoms of the disease; in small doses it confers immunity.

Should it be shown, even at an early day, that malignant growths are the result of microbial action, and may be destroyed, as the bacillus of anthrax, by their own products, we might see something hopeful, were it not that these products, if sufficiently active and abundant, are likewise noxious the host.

The evil bringing its own cure is the realization of the old superstition, according to which a viper's bite is cured by its own fat, and virtue found in the hair of the dog that bites. It may, indeed, be thought that the life products of every organism are injurious to itself. The atmosphere which man creates around him is fatal to him; a fish poisons its own element, and even plants render their soil unfit for their maintenance, not only by exhaustion, but, as some have thought, by excretion.

In the light of our present knowledge of the subject under discussion, we can do no better than proceed upon the theory that cancer is a disease of a local nature at first, becoming constitutional or general in its latter stages, and acting upon this theory, we must eradicate it completely, by removing everything well beyond all diseased tissue as quickly as possible.

How shall we best do this? The history of the operation of vaginal hysterectomy for this disease is, to me, exceedingly instructive, because of the one fact, if no other, of its astonishingly rapid development and exceedingly brilliant results secured in so short a period of time.

It is true that Soranos, of Ephesus, in his book on "Diseases of Women," published a century before Christ, speaks of the operation. It is also true, I believe, that Sauter, of Constance, in 182[illegible], successfully extirpated a cancerous uterus per vaginam, but it is almost certain that the operation had not been performed more than a half dozen times before the year 1879, when Czerney, struck with the report of Langenbeck's case reintroduced the operation with success. Billroth, Mikulicz, Scroeder, Hennig, Freund, Olshausen, Price, Tannen, Pean, Martin, Kaltenbach, Léopold, Bernays and others soon followed, and the operation now took its place among the established proceedings in surgery.

In October, 1889, when Greig-Smith completed the third edition of his great work on abdominal surgery, the operation had been performed about 380 times throughout the world. To-day we have the records of about 1,200 vaginal hsterectomies, and a mortality very little over 8 per cent, notwithstanding the operation is performed in all civilized countries, and, perhaps by untrained operators; and it is possible that the operation has suffered at the hands of these; yet, with a constantly improved technique, even better results will surely follow. Can more be said for even simple ovariotomy?

The absolute necessity of a diagnosis unequivocally correct cannot too much be insisted upon. For the operation contemplated, as all know, is one of the highest in the entire domain of surgery, and one upon which the issues of life and death depend. The beneficence of a successful operation is in proportion to the malignancy of the affection. If however after the ablation of the organ the condition of benignancy should be ascertained, there will be revealed the fact, that an operation attended with great hazard has been needlessly performed. The eclat consequent upon the performance of so grave an operation for a benign condition, will not, before the mental tribunal of the operator, atone for the remorse—consequent upon the consciousness of having inflicted an irreparable and inexcusable mutilation. I am disposed to agree fully with, and indeed adopt, the conclusion reached by Dr. J. F. Binnie of Kansas City, who recently contributed a valuable paper upon the subject of vaginal hysterectomy with, perhaps but one exception.

Dr. Binnie's conclusions are as follows:

1. Vaginal hysterectomy is a comparatively safe operation.

2. Many cases of vaginal-hysterectomy effect an absolute cure. Where it does do so it generally gives relief from distressing symptoms.

3. The more localized the cancer is, the sooner should its total extirpation be performed.

4. When it is even surmised, but not known positively that all the disease can not be removed, the operation ought to be performed.

5. Adhesion in the upper portion of the uterus, when there is cancer in the lower, call for, at least, an exploratory laparotomy; and if the adhesions prove to be the result of inflammation, hysterectomy may be performed.

6. Superficial extension of the disease over the vagina does not absolutely contra-indicate operation.

7. Clamps for the control of hæmorrhage are probably as safe as ligatures, if antiseptic precautions are rigorously attended to, certainly their application is infinitely easier and more rapid.

An exception to which I should refer is, the preference for the use of the clamps to control hæmorrhage over the ligature. The advantages to the patient by the use of the ligature are undoubtedly greater than by the use of the clamp. The ligature is more comfortable to the patient, and by it the danger of sepsis and sloughing is lessened; the healing process is consummated earlier; furthermoie, there is less risk of secondary bleeding and intestinal adhesions; and above all, the use of the ligature is more in harmony with a skillful surgical technique. The application of the clumsy clamp is, in my opinion, a coarse and unsurgical procedure.

To proceed step by step with the method which I employ.

The first may be designated as the Preparatory. The patient for several nights preceding the day of the operation, must have moderate doses of some favorite aperient, and on each day during the same period of time the parts contiguous to the seat of operation must receive a thorough cleansing. The vagina is irrigated twice daily with some trustworthy antiseptic, either solution of carbolic acid or corrosive sublimate. After irrigation, iodoform powder is to be insufflated, and a hedget of iodoform gauze is to be inserted, and removed for the next irrigation.

When the patient is placed ready for operation, a final and thorough douching with a strong antiseptic lotion should be instituted.

The bladder and the rectum are, of course, thoroughly emptied.

The best *position* of the *patient* is that of extreme perineal lithotomy, and the posture is best maintained by two strong and steady assistants.

*Fixation* and *manipulation* of the uterus is managed by means of a powerful vulsellum, with four broad interlocking teeth.

The uterus is now pulled down as far as possible by means of the vulsellum and then handed over to an assistant.

Dissection of the Vaginal Mucous Membrane of the Cervix. —at this stage lateral retractors may be applied for the purpose of giving more room and more light. A pair of scissors curved on the flat is made to cut through the mucous membrane around the cervix at a distance well clear of the disease.

A ligature is now carried around both uterine arteries upon either side by means of a blunt needle attached to a handle of sufficient length. This ligature must include also the tissues directly in contact with the artery, and much unnecessary bleeding will be avoided in consequence.

These arteries, together with the mass of tissue engaged by the ligature, are now divided by the scissors.

The uterus is now separated from all its attachments antero-posteriorly up to the broad ligaments by means of the finger exclusively. This being completed the retractors and the vulsellum are removed. The fundus of the uterus is now seized by means of the vulsellum from behind and drawn downward and outward, until ligatures can be applied to each broad ligament and ovarian arteries. These are now firmly tied, when the uterus is completely separated by means of the scissors from all its attachments and removed. The vagina is now thoroughly irrigated with warm water, and at intervals sponged out, in order to determine if there are any bleeding points. If appearances are satisfactory as they are likely to be, the vagina is tamponed with iodoform gauze, and the patient is at once removed to a bed ; a number of bottles of hot water applied to the surface of the body. No food whatever is allowed the patient for twenty-four hours.

Hitherto I have rehearsed history and mode, addressed to the ear ; evidences presented to the eye may augment the force of conviction. I have the pleasure of exhibiting an uterus affected with epithelioma. This the last uterus removed by the method detailed. It was obtained from a woman 58 years of age. This is the usual size of the organ in advanced life, after functional activity has ceased. Palpable signs of the malignant involvement are readily perceptible. The mucous surface of the vagina at the fornix, and the serous investment at the fundus, are distinctly visible also; as well as the cornua, the site of attachment of the Fallopian tubes and of the broad ligaments. After the total ablation of the uterus, and the arrest of the bleeding, the fenestrum, thus occasioned into the peritoneal cavity, was left without suture for purposes of drainage; and then the vagina was packed with iodoform gauze. The patient was then put to bed where she rallied well from the chloroform narcosis. The temperature at no time rose above 101 degrees. She made a rapid and uneventful recovery, and was removed to her home on the fourteenth day after the operation, bequeathing to me her uterus as a trophy, of the unspeakable beneficence of kolpohysterectomy.

